# Antibody-based alternatives to animal testing for toxin detection and antitoxin evaluation

**DOI:** 10.3389/ftox.2025.1636246

**Published:** 2025-09-09

**Authors:** Seo-Hyuk Chang, Jieun Jang, Wonjun Yang, Nam-Kyung Lee, Seoyeon Choi, Hyo-Il Jung, Wantae Kim, Boksik Cha, Sung-Jin Yoon, Ji-Yoon Noh, Jangwook Lee

**Affiliations:** ^1^ Biotherapeutics Translational Research Center, Korea Research Institute of Bioscience and Biotechnology, Daejeon, Republic of Korea; ^2^ Department of Biomolecular Science, Korea Research Institute of Bioscience and Biotechnology, School of Bioscience, Korea University of Science and Technology, Daejeon, Republic of Korea; ^3^ School of Mechanical Engineering, Yonsei University, Seoul, Republic of Korea; ^4^ TheDABOM Inc., Seoul, Republic of Korea; ^5^ Department of Integrated Medicine, Yonsei University, Seoul, Republic of Korea; ^6^ Department of Life Science, University of Seoul, Seoul, Republic of Korea; ^7^ Department of Physiology, Daegu Catholic University School of Medicine, Daegu, Republic of Korea; ^8^ Environmental Disease Research Center, Korea Research Institute of Bioscience and Biotechnology, Daejeon, Republic of Korea; ^9^ Department of Functional Genomics, Korea Research Institute of Bioscience and Biotechnology, School of Bioscience, Korea University of Science and Technology, Daejeon, Republic of Korea; ^10^ Aging Convergence Research Center, Korea Research Institute of Bioscience and Biotechnology, Daejeon, Republic of Korea

**Keywords:** antibody-based alternatives, toxin detection, antitoxin evaluation, animal replacement methods, 3Rs principle

## Abstract

Animal experiments have long played a central role in biomedical research and toxicology, yet their limitations in translational accuracy and ethical concerns have intensified the demand for reliable alternatives. Antibody-based technologies are versatile tools used to develop non-animal testing methods capable of detecting toxins and evaluating antitoxins. Enzyme-linked immunosorbent assay and lateral flow assays, among other techniques, have demonstrated high specificity, sensitivity, and reproducibility and are useful in diagnostics, therapeutic development, and as platforms to replace traditional animal assays. Recent developments in *in vitro* systems, including organoids and microphysiological systems, as well as the integration of AI-based *in silico* models, offer promising directions. Standardization and regulatory acceptance remain key challenges. A coordinated approach can facilitate the development of antibody-based systems to fulfill the goal of the 3Rs.

## 1 Introduction

Animal models, particularly rodents, have been vital in biomedical research and drug development. Indeed, these models have facilitated the elucidation of evolutionarily conserved biological mechanisms and the evaluation of pharmacological efficacy and toxicity prior to commencing human trials. Furthermore, since many aspects of human biology are inaccessible and due to ethical limitations in human studies, animal experiments have helped fill numerous knowledge gaps.

However, limitations of animal models in predicting clinical outcomes remain. For instance, over 150 clinical trials targeting inflammatory responses in critically ill patients have failed to yield effective therapies despite promising preclinical results in mice. Such discrepancies highlight the challenges posed by interspecies differences in immune function, metabolic pathways, and disease progression, thereby compromising translational accuracy. Nevertheless, several drugs have been developed and used in clinical settings following preclinical animal studies. Animal models remain critical for detecting safety issues and assessing the reversibility of toxic effects, factors not fully captured by *in vitro* systems.

Simultaneously, public and scientific concern over animal welfare has fueled the search for ethically and scientifically robust alternatives. The 3Rs principle, “Reduction, Refinement, and Replacement,” first proposed by Russell and Burch in 1959, continues to guide efforts in minimizing animal use. Advances in cell-based assays, computational toxicology, and high-throughput biochemical techniques have yielded a growing repertoire of non-animal approaches. Notably, several organizations, such as the European Centre for the Validation of Alternative Methods (ECVAM) and the U.S. National Institute of Environmental Health Sciences (NIEHS), have played a central role in validating and promoting these methodologies.

Antibodies, highly specific proteins produced by B cells, recognize and bind to target antigens with high affinity. Owing to their remarkable specificity and adaptability, antibodies have been utilized in various biomedical applications, including diagnostics, therapeutics, and toxin detection. In toxicology, antibody-based assays, such as ELISA and surface plasmon resonance, can identify and quantify toxins with high sensitivity and selectivity, providing a promising alternative to animal-based assays in research and regulatory contexts. This review aims to outline the development and application of antibody-based alternatives in toxin detection and antitoxin evaluation and discuss their scientific rationale, practical advantages, and ongoing challenges in validation and standardization.

## 2 Current status of alternatives to animal experiments

### 2.1 Regulatory progress and international adoption of non-animal test guidelines

Ethical imperatives, regulatory reforms, and scientific advancements have propelled the development and implementation of non-animal testing strategies. The introduction of the 3Rs principle has guided global efforts to minimize animal use in research and testing. In parallel, the wide array of *in vitro* and *in silico* methods has offered more human-relevant, cost-effective, and reproducible alternatives for toxicity assessment (Jin et al., 2020; Huang et al., 2021).

Regulatory agencies, such as the OECD, have also endorsed validated alternative test guidelines for endpoints, including skin sensitization, ocular irritation, and systemic toxicity (Corvaro et al., 2017). In the United States, the Interagency Coordinating Committee on the Validation of Alternative Methods (ICCVAM) has spearheaded the promotion of the regulatory acceptance of assays, such as the Bovine Corneal Opacity and Permeability (BCOP) and Isolated Chicken Eye (ICE) tests, recognized by the FDA, EPA, and CPSC, among other agencies. Legislation, such as the Lautenberg Chemical Safety Act in the U.S. and the REACH regulation in the European Union (EU), has mandated alternative methods where feasible.

More recently, the FDA Modernization Act 2.0 authorized the use of non-animal data for drug safety and efficacy evaluations, reinforcing the shift toward ‘New Approach Methodologies’ (NAMs) (Busquet et al., 2020). The EU has promoted the use of non-animal-derived antibodies due to their reproducibility and ethical advantages (Gray et al., 2020). The COVID-19 pandemic highlighted the limitations of traditional animal models in infectious disease research, accelerating global interest in alternatives. In this context, small organism models, including zebrafish (*Danio rerio*) and *Caenorhabditis elegans*, have gained attention (Gö ethel et al., 2022), and advanced techniques, such as microphysiological systems (MPSs), have not been widely adopted.

Despite substantial progress, regulatory integration of alternative methods remains challenging. Differences in national validation standards, lack of internationally harmonized protocols, and limited cross-species translation have hindered broader implementation. Therefore, continued global collaboration and investment in validation and standardization remain essential to achieving widespread regulatory acceptance of non-animal approaches.

### 2.2 Remaining roles of animal models in drug development

While non-animal alternatives continue to evolve, animal models retain a critical role in specific aspects of drug development. Rodents, particularly mice, have made significant contributions to understanding disease mechanisms and identifying therapeutic targets through evolutionarily conserved gene networks and regulatory pathways (Powell et al., 2022; Sullivan et al., 2023). Comparative genomic studies involving primate species have improved the interpretation of human disease variants (Gao et al., 2023). Integration of mouse phenotyping data with machine learning has enhanced the prediction of disease-relevant genes (Alghamdi et al., 2022).

Animal models are indispensable in pharmacokinetics (PK) research for evaluating absorption, distribution, metabolism, and excretion (ADME) properties. Rodent-based PK studies have helped define exposure–efficacy and exposure–toxicity relationships in early-phase development. Advances, such as microsampling, dried blood spot analysis, and physiologically based pharmacokinetic (PBPK) modeling, have enhanced efficiency and reduced animal use (Leblanc et al., 2018; Yuan et al., 2022). For instance, chimeric mice with humanized livers offer a better predictive system for human drug metabolism (Naritomi et al., 2019), and *in vivo* datasets continue to inform machine learning-based PK prediction models (Obrezanova et al., 2022).

Nonetheless, concerns persist regarding the limited capacity of animal models to replicate the complexity of human diseases. Methodological inconsistencies and interspecies differences frequently hinder effective clinical translation. Models for chronic conditions, such as Alzheimer’s disease or diabetic cardiomyopathy, have been criticized for failing to capture key pathological features, including aging or blood–brain barrier dynamics (Lezoualc’h et al., 2023; Padmanabhan and Götz, 2023). Similar limitations exist in cancer, pulmonary hypertension, and other disease models (Long et al., 2022; Wu X. H. et al., 2022; Sahara and Yanai, 2023), necessitating human-relevant, mechanism-reflective alternatives.

Despite these limitations, animal models remain indispensable at specific stages of drug development. Particularly in late-stage preclinical testing, they are crucial tools for assessing systemic toxicity, immune response, and overall pharmacodynamic behavior under complex physiological conditions. As regulatory authorities often require *in vivo* validation before clinical entry, animal models function as a transitional bridge between *in vitro* data and first-in-human studies. Therefore, they are not easily replaceable by current alternatives, highlighting the complementary nature of animal and non-animal methods.

### 2.3 Classic alternatives to animal testing

Several well-established *in vitro* and *ex vivo* test methods have been internationally recognized as replacements for traditional animal-based assays. For example, BCOP and ICE tests, using excised bovine or chicken eyes to assess corneal opacity and permeability, are OECD-approved alternatives for ocular irritation testing widely applied in regulatory safety evaluations, particularly in cosmetics and ophthalmic formulations. Although reliant on animal-derived tissues, they significantly reduce the need for live animal use and provide reproducible mechanistic data. Their limitations include reduced sensitivity to reversible or mild irritants, necessitating the integration of these tests into tiered testing strategies.

Skin sensitization assessment has advanced with the development of assays targeting key molecular events in the adverse outcome pathway (AOP) and is now supported by methods endorsed in the OECD TG 442 series. Representative examples include the Direct Peptide Reactivity Assay (DPRA), which evaluates the chemical reactivity of test substances through covalent binding to model peptides, KeratinoSens™, which measures transcriptional activation of the antioxidant response element (ARE) pathway in keratinocytes, and the human Cell Line Activation Test (h-CLAT), which assesses immune cell activation via the expression of CD86 and CD54 in dendritic cell-like lines. Though not antibody-based, these methods have contributed to substantial reductions in animal testing and are routinely used in defined approaches for hazard identification.

Receptor binding assays (RBAs), performed to quantify ligand-receptor interactions, are alternatives to *in vivo* toxicity assays that have replaced mouse bioassays in the detection of marine biotoxins, such as ciguatoxins and paralytic shellfish poisoning toxins (Ruberu et al., 2003; Hardison et al., 2016). Indeed, RBAs are extensively used in pharmacology, particularly in studies on GPCRs and nuclear receptors. While not inherently antibody-dependent, certain RBA platforms utilize antibody capture strategies; nonetheless, limitations such as poor mimicry of dynamic *in vivo* conditions and susceptibility to artifacts, such as non-specific binding and fluorescence quenching, remain to be addressed (Botana et al., 2010; Hulme and Trevethick, 2010; Breen et al., 2016).

Enzyme-linked immunosorbent assay (ELISA) remains a widely used method for evaluating immune responses and measuring antigen-specific antibody titers, especially in vaccine potency testing for diphtheria and tetanus (Jariyapan et al., 2017; De-Simone et al., 2023). ELISA’s potential for standardization and high-throughput capacity has made it broadly applicable in research and regulatory settings, with enhanced formats (e.g., biotin–streptavidin systems and antigen adsorption), improving sensitivity and reproducibility (Maple et al., 2001). However, ELISA has limitations in directly assessing neutralization efficacy and may show inter-laboratory variability and cross-reactivity (van Hoeven et al., 2008; Hifumi et al., 2014). In toxicology and pharmacology, classical alternatives, including organ-based assays, cell activation models, receptor binding assays, and ELISA, have reduced the reliance on animal testing. Nevertheless, classical methods may fall short in evaluating toxin potency or antitoxin activity with sufficient precision. Thus, advancing specialized, mechanism-based *in vitro* assays capable of replacing *in vivo* models in toxin and antitoxin evaluation remains necessary. In the following section, we discuss this need by exploring key toxin categories and emerging antibody-driven strategies for accurate detection and efficacy testing.

Most alternative approaches described herein are primarily utilized during the early stages of drug development, such as discovery, screening, or pilot studies. While these methods are effective in reducing animal use during initial phases, their application in later-stage confirmatory toxicology remains limited. In particular, animal studies are still required in the final stages of preclinical development to evaluate systemic toxicity, recovery from adverse effects, and whole-organism pharmacokinetics, all of which are critical aspects that have yet to be fully replicated by *in vitro* or *in silico* methods. Therefore, despite ongoing advancements, animal models remain indispensable for ensuring the safety of drug candidates prior to first-in-human trials.

## 3 Alternative models for toxin titer and antitoxin activities

Toxins are bioactive molecules produced by various organisms, including bacteria, fungi, plants, and animals. These compounds, ranging from proteins and peptides to small molecules, can induce severe physiological damage through various mechanisms, including interference with cellular signaling, disruption of target proteins, and immune activation (Dressler and Adib Saberi, 2005; Lindsay and Griffiths, 2013). Although some toxins have therapeutic value in controlled settings (e.g., botulinum toxin), most pose significant public health threats. Current diagnostic and therapeutic systems for toxin-related illnesses, such as botulism, diphtheria, and tetanus, rely heavily on animal models, including the mouse lethality assay and serum-derived antitoxins from immunized animals. These traditional approaches raise ethical concerns and are resource-intensive, necessitating the development of sensitive, scalable, and animal-free detection and evaluation methods.

Antibody-based *in vitro* platforms represent a promising alternative for quantifying toxin activity and assessing the neutralizing capacity of antitoxins.

### 3.1 Types of toxins and their origins

Toxins produced by various organisms can broadly be classified based on their biological origin, including bacterial, fungal, or animal-derived. Microorganisms in diverse environments produce a wide range of toxins (Rajkovic, 2014; Gupta et al., 2016; Nwaji et al., 2022). Bacterial toxins can be classified into endotoxins, which are released during bacterial lysis, and exotoxins, which are secreted into the external environment (Cavaillon, 2018). *Clostridium botulinum*, *Corynebacterium diphtheriae*, and *Staphylococcus aureus* produce potent exotoxins that severely impact human health (Lindsay and Griffiths, 2013; Cenciarelli et al., 2019; Wenzel et al., 2020). Botulinum neurotoxin (BoNT) from *Clostridium* species blocks neurotransmitter release, causing botulism and muscle paralysis. Their production and use are strictly regulated in many countries owing to the potential for bioterrorism (Cenciarelli et al., 2019; Janik et al., 2019). Staphylococcal enterotoxins (SEs), particularly SEB, are superantigens that bind to MHC class II molecules on antigen-presenting cells and T cell receptors, resulting in the release of high levels of cytokines and severe inflammation (Lindsay and Griffiths, 2013). Shiga toxin, produced by *Shigella dysenteriae* type 1 and enterohemorrhagic *Escherichia coli*, comprises two subunits: the A subunit binds to ribosomes, inhibiting protein synthesis and causing cell death, while the B subunit binds to the globotriaosylceramide receptor on the cell membrane to facilitate cellular entry (Gyles, 2007; Melton-Celsa, 2014). Exposure to Shiga toxin can lead to diarrhea, fever, and vomiting, and, in severe cases, hemolytic uremic syndrome (Obrig, 2010; Keir et al., 2012).

Fungi produce mycotoxins, secondary metabolites synthesized by genera such as *Aspergillus*, *Penicillium*, and *Fusarium* (Abraham et al., 2022). These toxins include aflatoxins, ochratoxins, and zearalenone, which are associated with liver and kidney toxicity and, in some cases, carcinogenicity (Reddy et al., 2010; Claeys et al., 2020). Aflatoxin B1, produced by *Aspergillus flavus* and *A. parasiticus*, is among the most potent carcinogens commonly found in contaminated grains and nuts (Awuchi et al., 2020). It is classified as a Group 1 carcinogen by the International Agency for Research on Cancer (Wogan et al., 2012). Consequently, many regulatory agencies have established strict detection standards to mitigate potential public health risks (Robens and Cardwell, 2003; Wu and Guclu, 2012). Zootoxins, produced by insects, arachnids, reptiles, and marine organisms, pose significant health risks (Nwaji et al., 2022). These toxins act through various mechanisms, including inflammation, neurotoxicity, coagulopathy, and cytolysis (Guido-Patiño and Plisson, 2022). Common toxin-producing animals include ants, wasps, scorpions, spiders, snakes, algae, and shellfish (Garthwaite, 2000; Kularatne and Senanayake, 2014).

Most antitoxins and antivenoms are polyclonal antibodies derived from the sera of toxin- or venom-immunized animals. While effective, these treatments may induce acute immunogenic responses and lack cross-reactivity to different toxin variants (Dixit et al., 2016). Furthermore, their production and potency testing depend on animal use, further raising ethical and technical challenges. The development of *in vitro* assays and antibody-based detection platforms to replace animal-based systems for antitoxin production and evaluation has gained traction (Mukherjee et al., 2012; Doke and Dhawale, 2015; Akkermans et al., 2020).

### 3.2 Conventional methods to detect toxins or evaluate antitoxin activity

#### 3.2.1 Biological methods

##### 3.2.1.1 Animal lethality assay: the gold standard

Animal testing has traditionally been regarded as the “gold standard” for evaluating the toxicity of chemicals and the potency of antitoxins. Animal models are widely used due to their physiological similarities to humans, allowing for data extrapolation to human systems with a certain degree of confidence (Akhtar, 2015). In standard toxicity assays, animals, including mice, rats, rabbits, dogs, and cats, are administered toxins to observe physiological responses and lethal effects (Roffey et al., 2003). In antitoxin potency tests, toxins are pre-incubated with candidate antitoxins before administration to animals, and survival rates are measured (Lindstrom and Korkeala, 2006).

While these models have contributed to our understanding of toxicodynamics and are essential in regulatory safety assessments, they face ethical challenges and translational limitations. With growing public awareness and scientific concern for animal welfare, there has been an urgent need to develop alternative methods that can reduce, refine, or replace the use of animals in toxicology testing (Balls, 1991; Doke and Dhawale, 2015). In response, various *in vitro*, *in silico*, and tissue culture-based approaches have been actively explored for decades (Reverté et al., 2014; Caloni et al., 2022; Guarra and Colombo, 2023).

##### 3.2.1.2 Cell or tissue culture assay

Various cell-based methods have been developed using either primary cells derived from tissues or immortalized cell lines to replace animal-based assays (Banerjee and Bhunia, 2009; 2010; Reverté et al., 2014). In these assays, cells are treated with test substances, and viability is assessed using colorimetric or enzymatic methods, such as the MTT or LDH assays (Fotakis and Timbrell, 2006). These *in vitro* systems enable high-throughput screening and are cost-effective and ethically favorable, although lack the complexity of whole-organism responses. Three-dimensional (3D) organoids and organ-on-a-chip technologies have been introduced to better recapitulate physiological tissue interactions, overcoming the aforementioned limitation (Forsythe et al., 2018; Picollet-D’hahan et al., 2021).

In the case of BoNTs, the current therapeutic standard is equine-derived antitoxin, and its efficacy has been evaluated using mouse bioassay (Rao, 2021). However, alternatives have been investigated; for example, the neutralizing activity of BoNT/E antitoxins has been evaluated using the human neuroblastoma SiMa cell line (Ben David et al., 2022). Since BoNT/E cleaves synaptosomal-associated protein (SNAP-25, 25 kDa) (Binz et al., 1994), cleaved SNAP-25 has been used as a surrogate marker of BoNT activity *in vitro*. A high correlation with *in vivo* mouse bioassays suggests that such cell-based systems have the potential to replace animal models.

Similar efforts have been made in the development of snake antivenom, where a single-chain variable fragment (scFv) antibody was generated against the cytotoxin in *Naja atra* venom, and its neutralizing capacity was assessed in C2C12 muscle cells (Liu et al., 2022). scFv has been shown to significantly neutralize venom-induced cytotoxicity, providing an example of *in vitro* screening for antivenoms that avoids animal use. *In vitro* cell-based assays have been explored to evaluate the potency of antitoxins against tetanus toxoid, SEB, and mycotoxins (Xia et al., 2017; Rasooly et al., 2018; Ticha et al., 2021).

##### 3.2.1.3 Protein interaction assay

Another strategy for evaluating toxicity involves protein–protein interaction (PPI) assays, which examine the binding affinity of a toxin to its cellular target or how antitoxins disrupt these interactions (Frisch et al., 2003; Lindsay and Griffiths, 2013; Lite et al., 2020). These assays provide mechanistic insights into how toxins exert their biological effects and neutralizing agents interfere with such processes (Thakur et al., 2017; Shi et al., 2023). For example, a competitive binding assay has been developed to evaluate antivenom activity against neurotoxic venom from elapid snakes (Pruksaphon et al., 2020). Herein, microplates are coated with a nicotinic acetylcholine receptor (nAChR) ligand, NK3, and the ability of venom to inhibit nAChR–ligand interaction is measured. Antivenom-mediated restoration of binding is used as a surrogate to assess neutralization efficacy. This *in vitro* system strongly correlates with animal lethality assays, indicating its potential as a non-animal alternative. Similar PPI-based detection systems have been developed for toxins, including Shiga toxin, BoNT, and SEB (Tallent et al., 2013; Wild et al., 2016; Li et al., 2024).

#### 3.2.2 Biochemical methods

##### 3.2.2.1 Polymerase chain reaction (PCR)-based detection

PCR is a well-established molecular technique used to detect toxins or toxin-producing organisms by amplifying specific DNA sequences (Persson et al., 2008; Alahi and Mukhopadhyay, 2017). This method enables the identification of bacterial species based on their toxin genes or the quantification of gene expression related to toxin production. A recent study targeting *Clostridioides difficile*, a common cause of antibiotic-associated diarrhea, developed a multiplex real-time PCR assay capable of directly detecting toxigenic genes from fecal samples without requiring bacterial culture (Bagdasarian et al., 2015; Nagy, 2018). Unlike conventional methods that rely on culture followed by PCR or ELISA, this approach enables rapid and simultaneous detection of toxin genes, enhancing diagnostic efficiency. Due to its high sensitivity and flexibility in primer design, PCR has been widely used to detect a broad range of toxin-producing bacteria and fungi (Mclauchlin et al., 2000; Sadhasivam et al., 2017).

##### 3.2.2.2 Immunoassay

Owing to its high sensitivity and specificity, ELISA is a widely used immunological technique for detecting and quantifying toxins (Lequin, 2005; Hayrapetyan et al., 2023). ELISA is routinely applied in food safety, clinical diagnostics, and pharmaceutical testing to identify target antigens using specific antibodies (Mikulskis et al., 2011; Iha et al., 2019; Xiong et al., 2020).

The assay’s performance depends heavily on the quality of the antibodies; therefore, antibody engineering, signal amplification, and advanced detection platforms have been employed to improve sensitivity (Peng et al., 2022). ELISA is a potential alternative to animal testing in evaluating the potency of tetanus toxoid vaccines. Iwaki et al. (2023) reported a competitive ELISA in which tetanus toxoid-coated microplates were incubated with anti-tetanus antibodies and serum from immunized mice. The competition between serum antibodies and reference antibodies has been quantified, showing over 95% correlation with traditional *in vivo* assays. Thus, ELISA-based methods can reduce animal use in potency testing while maintaining reliability. Although ELISA is a well-established method with high reproducibility, variations in assay formats, reference materials, and validation procedures across laboratories remain barriers to regulatory acceptance that must be addressed for the broader implementation in standardized testing.

##### 3.2.2.3 Lateral flow assays (LFAs)

LFA is a portable immunoassay that enables rapid, on-site detection of toxins, pathogens, or analytes within 30 min (Koczula and Gallotta, 2016). Using membrane-based strips embedded with antibodies specific to the target, LFAs are widely used in point-of-care settings due to their simplicity, low cost, and visual readout (Butler et al., 2001; Shyu et al., 2002; Morales-Narváez et al., 2015). However, the trade-off for portability is lower sensitivity and limited quantification capacity compared to laboratory-based assays (Deng et al., 2021).

**FIGURE 1:**
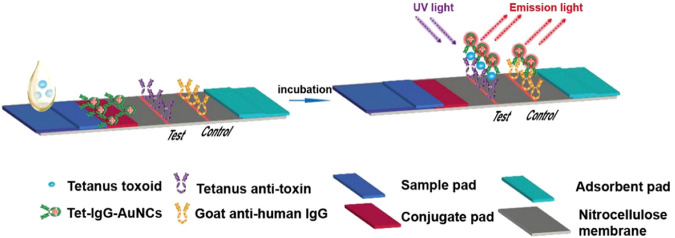
Schematic representation of a fluorescent immunochromatography test strip for detecting tetanus toxoid. The assay utilizes tetanus toxoid antigens labeled with gold nanoclusters (Tet-IgG-AuNCs) as the detection probe. Upon sample application and incubation, anti-tetanus antibodies in the sample bind to the tetanus toxoid probes, which are captured at the test line by immobilized anti-toxin antibodies. Emission light is detected under UV excitation. The control line is coated with goat anti-human IgG to confirm the test validity. The LFA comprises a sample pad, conjugate pad, nitrocellulose membrane with test/control lines, and an absorbent pad. Figure adapted with permission from Elsevier (Zhuang et al., 2021).

**FIGURE 2:**
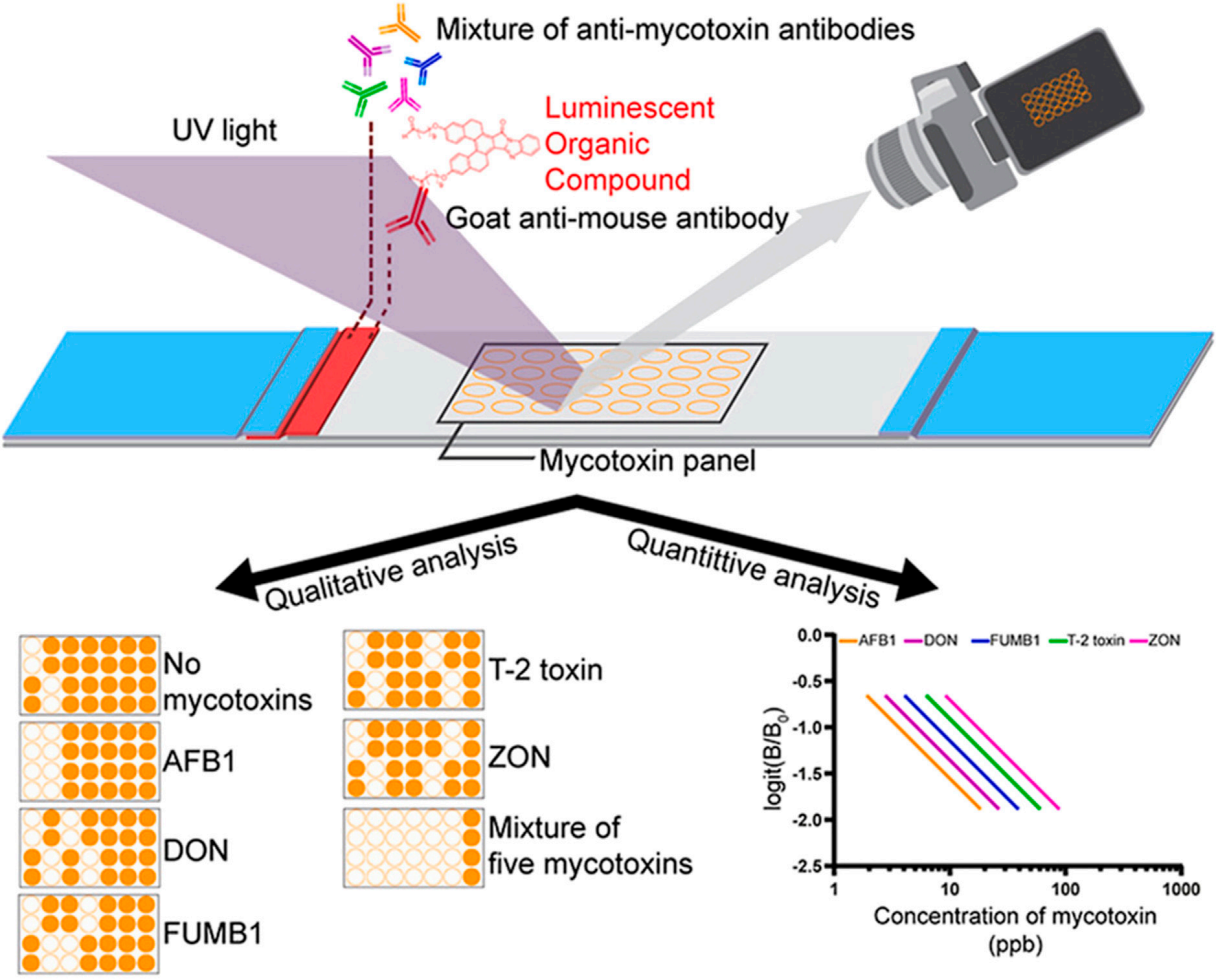
Microarray lateral flow strip test (μLFIA) for multiplex detection of five mycotoxins. A mixture of anti-mycotoxin antibodies and a novel luminescent dye (M424) conjugated with goat anti-mouse antibody was used as the reporter system. The mycotoxin panel contained AFB1, DON, FUMB1, T-2, and ZON. Signals were visualized under UV light, allowing both qualitative analysis (presence/absence of specific toxins) and quantitative analysis based on calibration curves. Representative results show detection of single and multiple mycotoxins, with logit(B/B0) plotted against concentration to construct standard curves for each toxin. Figure adapted with permission from Elsevier (Charlermroj et al., 2021).

**FIGURE 3:**
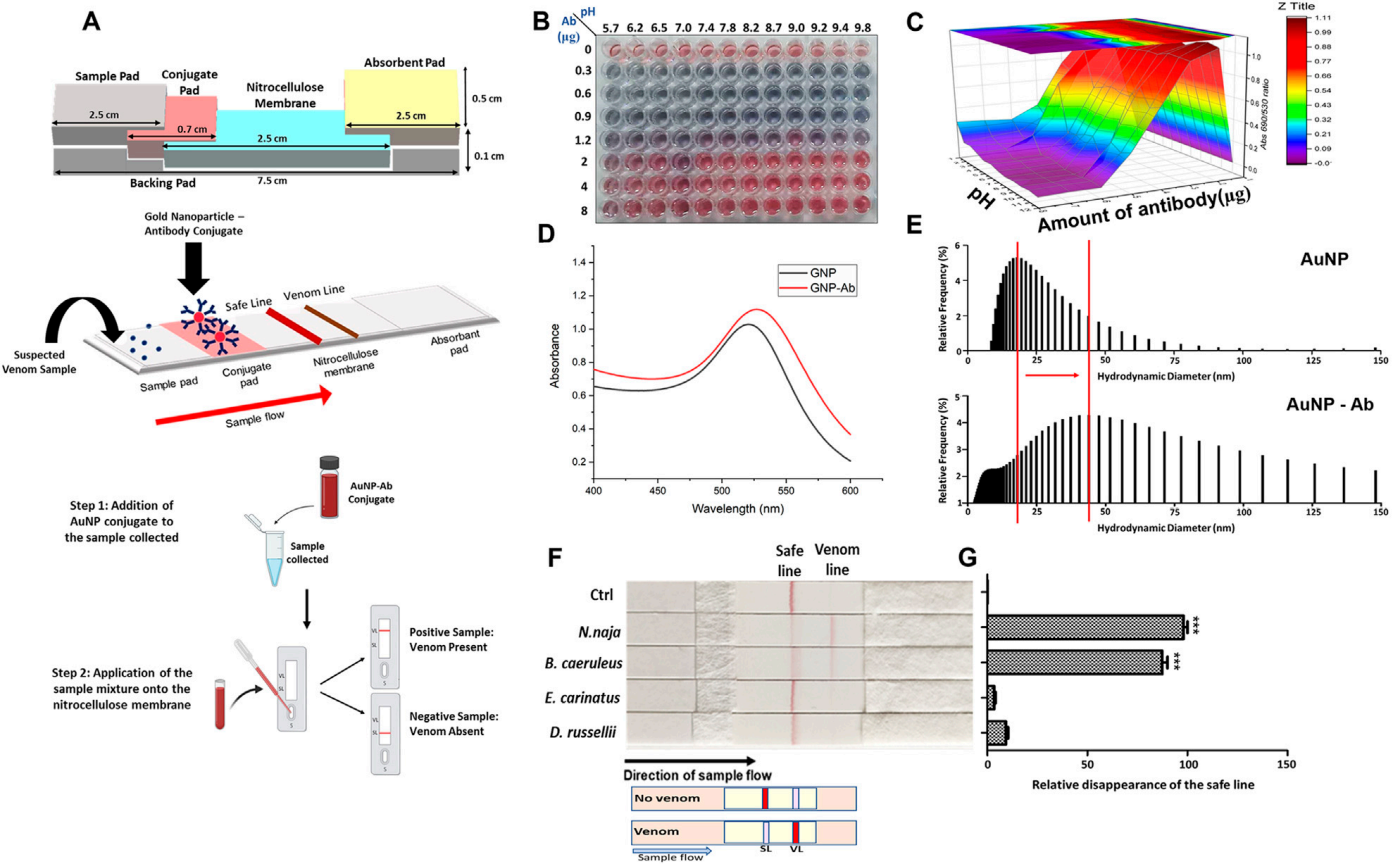
Antibody-based lateral flow assay (LFA) for detecting elapid venoms (Naja naja and Bungarus caeruleus). The assay employs gold nanoparticle–antibody (AuNP–Ab) conjugates for rapid and field-deployable detection. **(A)** Schematic representation of the assembled LFA strip, including sample pad, conjugate pad, nitrocellulose membrane, and absorbent pad, along with the AuNP–Ab conjugation principle. **(B)** Optimization of AuNP–Ab conjugation under varying pH and antibody concentrations, visualized by colorimetric change. **(C)** 3D surface plot depicting conjugation efficiency as a function of pH and antibody concentration. **(D)** UV–visible spectra comparing absorbance profiles of unconjugated AuNPs and AuNP–Ab conjugates. **(E)** Dynamic light scattering histograms showing the hydrodynamic diameter distribution of AuNPs before and after antibody conjugation. **(F)** Validation of the LFA strips with venoms from the “big four” snakes, demonstrating disappearance of the safe line upon testing with elapid venoms (Naja naja and Bungarus caeruleus), but not with viperid venoms (Echis carinatus and Daboia russelii). **(G)** Quantitative analysis of safe line disappearance, confirming significant responses for elapid venoms. Together, these results demonstrate the applicability of antibody-based LFAs in snakebite diagnostics. Figure reproduced from PLOS Neglected Tropical Diseases (CC BY license) (Kaul et al., 2021).

Due to their field-deployable nature, LFAs are widely applied for diverse diagnostic purposes. For example, an antibody-based fluorescent immunochromatography platform for tetanus toxoid detection has been developed, demonstrating its potential as a sensitive and animal-free alternative for potency assessment (Zhuang et al., 2021). This representative approach is illustrated in Figure 1. LFAs are also particularly suitable for rapidly detecting mycotoxins in grain and animal feed. A multiplex LFA capable of detecting five major mycotoxins, namely aflatoxin B1 (AFB1), deoxynivalenol (DON), fumonisin B1 (FUMB1), T-2 toxin (T-2), and zearalenone (ZON), has been recently developed by printing specific antibodies in an array format on the test strip, as illustrated in Figure 2 (Charlermroj et al., 2021). A novel fluorescent organic compound is used as a reporter, allowing for the sensitive detection of compounds in complex samples. This single-strip multiplex LFA enables simultaneous, rapid analysis of multiple toxins in a single run, providing a valuable tool for food safety monitoring. A representative example of such an advanced LFA system is illustrated in Figure 3 (Kaul et al., 2021). While LFA is rapid and simple, the absence of harmonized protocols and validated reference standards limits its regulatory acceptance. These challenges must be addressed to ensure consistent performance across different settings.

##### 3.2.2.4 Biosensors

Biosensors combine biological recognition elements with physicochemical detectors to facilitate real-time, label-free detection of toxins (Bhalla et al., 2016). These systems have high sensitivity and selectivity and are employed in environmental monitoring, food safety, and clinical applications (Van Dorst et al., 2010; Gavrilaş et al., 2022; Lino et al., 2022). One widely adopted biosensor is the surface plasmon resonance (SPR) platform, which detects changes in the refractive index on a metal-coated surface when biomolecules interact (Douzi, 2017). Unlike conventional assays, SPR does not require labeling or chemical modification of samples. An SPR-based sensor using a molecularly imprinted polymer specific to aflatoxin B1 (AFB1) has achieved detection limits as low as 1 pg/mL. The sensor demonstrated high specificity for AFB1 even in complex food matrices, such as peanuts and corn (Akgönüllü et al., 2020). Bio-layer interferometry (BLI) is another label-free biosensing technique that monitors interference patterns caused by molecular binding on the surface of a sensor tip (Shah and Duncan, 2014).

A BLI-based nanosensor has been designed using peptide aptamers specific to Shiga toxins, Stx1, and Stx2, with detection limits of 44.5 and 41.3 pg/mL, respectively (Kaur et al., 2020). These optical biosensors offer promising platforms for rapid, accurate, and animal-free detection of biologically active toxins. Biosensors have been limited by the lack of standardized calibration methods and regulatory guidelines. Developing robust reference frameworks is essential for their translation into routine testing.

### 3.3 Antibodies for alternative toxin detection methods

Accurate and sensitive detection of toxins is essential for effective diagnosis, surveillance, and therapeutic evaluation. Although PCR enables the identification of pathogenic bacteria by targeting specific DNA sequences (Persson et al., 2008; Alahi and Mukhopadhyay, 2017), it cannot distinguish whether the detected nucleic acids originate from live or dead organisms, which can lead to false positives. Additionally, factors like low-quality samples or nonspecific primer binding can further compromise accuracy (Fevraleva et al., 2022). Moreover, PCR does not provide direct information on the presence or activity of toxic proteins, thereby limiting its utility in assessing actual toxicity.

Complementary methods have been developed to overcome these challenges. Techniques, such as high-performance liquid chromatography or mass spectrometry, provide high-resolution chemical analysis but require costly equipment, trained personnel, and lengthy procedures (Banerjee and Bhunia, 2010; Hickert et al., 2015; Féraudet Tarisse et al., 2021). In contrast, immunoassays using antibodies provide an efficient and sensitive platform for detecting toxins and evaluating antitoxin efficacy.

Antibody-based immunoassays are relatively simple to perform, require minimal sample preparation, and can be standardized across laboratories. ELISAs, for instance, are highly reproducible and allow high-throughput screening of samples. Miniaturized formats, such as LFAs, enable rapid and on-site testing. Immunoassays can be adapted for use with optical and electrochemical biosensors, providing enhanced sensitivity through signal amplification and device integration (Weng et al., 2016; Li et al., 2021). Hence, antibody-based immunoassays are considered suitable alternatives to animal-based assays for detecting toxins and assessing the neutralizing efficacy of antitoxins. Regulatory authorities, including the US Food and Drug Administration (FDA), European Centre for Disease Prevention and Control (ECDC), and World Health Organization (WHO), have incorporated immunoassays into their approved detection frameworks. For example, the U.S. has approved PCR-based detection of the Shiga toxin genes (*Stx1* and *Stx2*) and commercial ELISA kits for detecting Shiga toxin-producing *E. coli* (STEC) (Gould et al., 2022; He et al., 2013). Meanwhile, the ECDC permits the use of cultures, PCR, and STEC-specific ELISA for confirmatory diagnostics. Similarly, *Clostridioides difficile* can be diagnosed using PCR and ELISA following fecal culture (McDonald et al., 2018).

Although significant progress has been made, toxins such as BoNTs are routinely tested using animal models, with the mouse bioassay considered the benchmark for BoNT detection (Hobbs et al., 2019). Similarly, evaluating the antivenom potency of venom toxins, particularly those derived from snakes, relies heavily on *in vivo* lethality testing due to their complex and variable composition. These assays necessitate a large number of animals and present challenges related to reproducibility and mechanistic insights. Consequently, continued efforts are needed to develop and validate antibody-based *in vitro* alternatives that offer equivalent or superior sensitivity, reproducibility, and functional relevance for bacterial- and animal-derived toxins.

Since many toxins exist in multiple isomeric forms, detection antibodies must recognize all clinically or environmentally relevant forms to avoid underestimating toxin levels and misinterpreting associated risks. Accordingly, selecting and validating antibodies requires comprehensive cross-reactivity testing with all relevant toxin isomers to ensure accurate measurement.

## 4 Antibodies as core reagents for animal replacement testing

Antibodies are essential for non-animal toxicity testing due to their high specificity, affinity, and adaptability across multiple assay platforms (Rucker et al., 2005; Zhu et al., 2014). They enable accurate and reproducible detection of toxins and assessment of antitoxin potency, supporting standardized endpoints for regulatory and high-throughput applications (Akgönüllü et al., 2020; Iwaki et al., 2023). Antibodies can be produced via polyclonal, monoclonal, or recombinant production methods, each varying in reliability, scalability, and ethical considerations (Leenaars and Hendriksen, 2005; Frenzel et al., 2013; Pedrioli and Oxenius, 2021). Recombinant approaches allow animal-free production with improved affinity, reduced cross-reactivity, and flexible assay compatibility (Hoogenboom, 2005; Schirrmann et al., 2011).

Immunoassay platforms, such as ELISA, LFAs, and SPR, leverage antibody–antigen interactions to yield sensitive quantitative results in complex biological samples (Lequin, 2005; Peng et al., 2022). ELISA is widely used to measure antigen-specific antibody titers, vaccine potency, and batch-to-batch consistency (Folegatti et al., 2020). Meanwhile, SPR enables label-free, real-time kinetic analysis that correlates with neutralization capacity in antitoxin testing (Brand et al., 2012; Quintilio et al., 2019). Both methods align closely with conventional *in vivo* assays, supporting their reliability as animal-free alternatives (Lassaunière et al., 2020). By optimizing antibody reagents and assay formats, these technologies facilitate the replacement of animal-based bioassays without compromising accuracy, sensitivity, or reproducibility in toxin detection and safety evaluation.

### 4.1 Detection antibodies for toxin titer measurement

Many natural toxins can cause significant biological damage even at extremely low concentrations, necessitating the development of highly sensitive detection systems. Among the available technologies, antibody-based assays, such as ELISA, are widely used to identify and quantify bacterial and venom toxins. These assays offer a rapid and specific means of detection, often replacing or complementing traditional culture methods or *in vivo* bioassays (Peng et al., 2022). Table 1 summarizes representative antibody-based detection assays and their applications in toxin titer measurement.

**TABLE 1:** Representative antibody-based diagnostic kits for toxin detection.

Toxin Type	Target Toxin	Antibody Type	Detection Format	Detection Limit	References/Application
Bacterial toxin	Botulinum neurotoxin (BoNT/A)	Monoclonal/Polyclonal	ELISA/SPR/Bead-based	pg/mL to ng/mL	Kalb et al. (2015), Nepal and Jeong (2020)
	Diphtheria toxin	Monoclonal	ELISA	<1 ng/mL	Zasada et al. (2013)
	Staphylococcal enterotoxins (SEA–G)	Monoclonal/Goat-derived	Hydrogel Biochip/ELISA	0.1–0.5 ng/mL	Poli et al. (2002a), Rubina et al. (2010)
Fungal toxin (mycotoxin)	Aflatoxin B1	Monoclonal	Lateral Flow Assay (LFA)	1–10 ng/mL	Pöhlmann and Elßner (2020)
	Zearalenone	Recombinant antibody	Multiplex LFA/ELISA	<5 ng/mL	Gdoura et al. (2022)
Venom toxin	Cytotoxin-7 (cobra, krait venom)	Monoclonal (AB1)	LFA/SPR	28.7–110 ng/μL	Kaul et al. (2021)
	Bungarus multicinctus venom	Rabbit polyclonal	ELISA/LFA	0.1–1 ng/mL	Nong et al. (2023)
	Taiwan neurotoxic/hemorrhagic venom	Species-specific monoclonals	LFA/ELISA	0.38–0.75 ng/mL (ELISA)	Liu et al. (2018)

#### 4.1.1 Bacterial toxins

Bacterial toxins pose significant public health risks due to their presence and pathogenicity. Antibodies can detect bacteria and their toxins in environmental, food, and clinical samples. BoNT is highly toxic and has therapeutic applications (Chen, 2012). Due to its low LD_50_ in mice, highly sensitive antibodies have been developed and incorporated into mass spectrometry-based immunocapture systems, magnetic bead-based assays, and nanosensors for BoNT detection (Poras et al., 2009; Kalb et al., 2015; Cheng and Chuang, 2019).

*Staphylococcus aureus* produces enterotoxins (SEs) that are associated with foodborne illness and toxic shock. Antibody-based immunoassays targeting SEs offer rapid and specific detection. For example, a sandwich ELISA can detect SEA and SEB at 0.1 ng/mL in human urine samples using polyclonal antibodies derived from goat serum. Additionally, hydrogel-based biochips can simultaneously detect seven SE serotypes within 0.1–0.5 ng/mL (Rubina et al., 2010). SE-targeted immunoassays have also been developed that incorporate fluorescent dyes, nanoparticles, and SPR-based methods to improve sensitivity and usability (Wu et al., 2016).

A comparative analysis of seven commercial antibody-based ELISA kits for anti-diphtheria IgG achieved reproducible results in 72 serum samples. However, discrepancies were observed in the measured values depending on whether the manufacturer’s instructions or the International Standard for Diphtheria Antitoxin were followed for calibration (Zasada et al., 2013). These discrepancies likely stemmed from differences in antigen and antibody qualities, as well as variations in buffer composition and blocking strategies, emphasizing the need for standardization.

#### 4.1.2 Venom toxin

Snakebite envenomation is a significant medical issue worldwide, and current antivenom production and quality control are primarily dependent on animal-based methods (Pucca et al., 2019). Several antibody-based LFA and ELISA systems have been developed as alternative methods for venom detection. One example is an LFA test targeting cytotoxin-7, a component found in elapid snake venom, which detects venom from four major medically relevant elapids in India, as illustrated in Figure 3 (Kaul et al., 2021). Mice were immunized with recombinant cytotoxin-7, resulting in the generation of a monoclonal antibody (AB1) that specifically recognizes cytotoxin-7 from cobra and krait venom. SPR analysis indicated AB1 affinities for recombinant cytotoxin-7, cobra, and krait venom at 31 nM, 311 nM, and 149 nM, respectively. The developed LFA achieved limits of quantitation (LoQ) of 28.7 ng/μL and 110 ng/μL for cobra and krait venom in spiked serum, respectively.

Another group developed an LFA to distinguish between hemorrhagic and neurotoxic venoms from snakes prevalent in Taiwan (Liu et al., 2018). Species-specific antibodies were generated by purifying immunoglobulins from hemorrhagic and neurotoxic antivenoms using Sepharose-immobilized venom proteins. The selected antibodies were incorporated into an LFA strip to distinguish venom types. The test’s LoQ was 0.38 ng/mL for hemorrhagic and 0.75 ng/mL for neurotoxic venoms in ELISA, and 5 ng/mL and 50 ng/mL, respectively, in LFA within 15 min. Specificity and sensitivity were 100% for neurotoxic venom but only 36% for hemorrhagic venom, highlighting limitations for broader application.

A new ELISA and LFA system can rapidly detect *Bungarus multicinctus* venom in blood and urine from envenomed animal models, demonstrating suitability for rapid field diagnosis (Nong et al., 2023). Rabbits immunized with BM venom produced species-specific IgG, purified through affinity chromatography with venom from other snake species. The resulting kits achieved detection limits of 0.1 ng/mL (ELISA) and 1 ng/mL (LFA).

These studies collectively highlight the utility of antibody-based detection systems to quantify toxin concentrations and support their use as reliable, animal-free tools for diagnostic and safety evaluation purposes.

### 4.2 Animal replacement assessment of anti-toxins

Antitoxin potency for bacterial diseases, such as botulism, tetanus, and diphtheria, has traditionally been assessed using mouse lethality assays. Animal-free alternatives, including ELISA-based platforms and cell-based functional assays, have been developed as ethically compliant and mechanistically relevant options (Bak et al., 2017; Torgeman et al., 2017). For instance, neuronal cell lines are utilized to evaluate botulinum antitoxins by quantifying cleaved SNAP-25, a BoNT/A-specific intracellular target. This method demonstrates strong reproducibility and alignment with *in vivo* data, highlighting its suitability for replacing animal tests in potency assessment. Similarly, antitoxin potency for tetanus and diphtheria has historically been evaluated in toxin-challenged animal models. However, ongoing efforts aim to replace such assays with *in vitro* methods, including immunoassays targeting specific epitopes or functional domains of the toxins (Manghi et al., 1994; Zhuang et al., 2021; Chen et al., 2022). These strategies result in reduced variability, decreased costs, and greater ethical acceptability.

Animal models have traditionally been utilized to determine the neutralization efficacy of antivenoms. However, these methods present substantial ethical and logistical challenges, including the substantial number of animals required and the variability of venom composition across species and regions (Pucca et al., 2019). To address these concerns, ELISA platforms have been developed to evaluate the binding and neutralization potential of antivenoms using purified venom components. One such system employs β-bungarotoxin from *B. multicinctus* as the antigen to test 55 equine plasma samples with established *in vivo* efficacy, yielding 97% sensitivity and 93% specificity even in the presence of competing toxic PLA2 enzymes (Liu et al., 2024). These results indicate that ELISA-based approaches can serve as effective surrogates for animal testing in antivenom evaluation.

In addition to ELISA, alternative *in vitro* methodologies—including enzymatic activity assays and PPI platforms—are being investigated to capture broader aspects of venom neutralization (Gutiérrez et al., 2017; Liu et al., 2024). The complex composition of snake venoms, featuring diverse proteins and enzymes, such as phospholipase A2 (PLA2), snake venom metalloproteinases (SVMPs), and matrix metalloproteinases (MMPs), presents significant challenges for developing standardized universal *in vitro* assays (Warrell, 2010; Calvete, 2011). Consequently, single-antigen assays often fail to capture the full spectrum of toxic activities. To achieve an accurate assessment of antivenom efficacy, an integrated approach employing cell-based models, receptor-binding studies, and multiplexed antigen panels may be required.

An emerging promising strategy involves the use of multivalent or multispecific antibodies capable of simultaneously targeting multiple toxin epitopes or components. This methodology has been shown to increase therapeutic efficacy in both *in vitro* and *in vivo* models. For instance, a bispecific antibody targeting two distinct pertussis toxin epitopes showed more than a two-fold increase in neutralizing activity compared to its monospecific counterparts (Wagner et al., 2016). Similarly, a bispecific antibody that binds to the surface exopolysaccharide Psl and the PcrV proteins of the type III secretion system sis currently being evaluated in clinical trials for *Pseudomonas aeruginosa* (DiGiandomenico et al., 2014). These developments suggest that advances in antibody engineering—particularly the development of bispecific or multispecific formats—are likely to improve the accuracy and standardization of animal-free platforms for assessing antitoxins and antivenoms.

## 5 Advanced methods supporting antibody-based alternatives

Recent biotechnology advances have led to the development of complex *in vitro* models (CIVMs), including 3D organoids, human-induced pluripotent stem cell-derived tissue constructs, and MPSs such as organ-on-a-chip platforms (Baran et al., 2022). These systems more accurately replicate human physiology and may replace animal tests in toxin and drug assessments by incorporating biomechanical stimuli, perfusion dynamics, and immune cell interactions.

Human intestinal organoids have been utilized in preclinical research to evaluate inhibitors of cholera toxin and *Clostridioides difficile* toxins (Zomer-van Ommen et al., 2016; Gonzales-Luna et al., 2023). However, areas like venom research continue to face challenges due to the limited understanding of the genomic and cellular underpinnings of toxin production. Evidence suggests that combining CIVMs and computation tools with antibody-based systems may help address these gaps.

### 5.1 Complex *In vitro* platforms to evaluate toxins

CIVMs, including organoids and MPSs, enable the assessment of human-specific physiological responses under controlled experimental conditions. These models replicate certain features of tissue architecture, perfusion, and immune interactions that are often lacking in conventional 2D cell cultures. When coupled with antibodies that bind to specific toxins for quantification, CIVMs serve as an effective platform for toxin detection, mechanistic studies, and neutralization screening.

Lung- and liver-on-a-chip models can be used to evaluate drug-induced toxicity and immunological responses, while brain-on-a-chip systems are increasingly utilized to model neurotoxicity and infectious challenges (Baran et al., 2022). These systems facilitate precise kinetic measurements and multiplex immune or signaling pathway readouts, which are valuable for assessing toxin-antibody interactions in biologically relevant contexts without relying on animals.

### 5.2 Venom organoids and genomic tools

Snake venoms pose unique challenges due to their complex composition and limited genetic characterization. Most venom-encoding genes and toxin-producing cell types remain poorly understood, which has limited progress in recombinant antivenom production and non-animal test development. To address this issue, a high-quality reference genome for the Indian cobra has been published, identifying 139 toxin genes across 33 families (Suryamohan et al., 2020). This genomic resource enables the design of recombinant antigens and antibody targets.

Additionally, venom gland organoids derived from adult stem cells can secrete functionally active toxins *in vitro* (Post et al., 2020; Alafeef et al., 2021). These long-term expandable cultures offer a human-relevant, scalable alternative to venom extraction from live animals, serving as a novel tool for studying venom biology and testing neutralizing antibodies under controlled laboratory conditions.

### 5.3 *In silico* and AI-powered models for antibody integration

Advances in AI and *in silico* modeling now allow rapid simulation of toxicological effects, antibody optimization, and reduced reliance on animal testing. Leveraging extensive biological datasets and machine learning algorithms, these methods can predict toxicity, immune responses, and antigen–antibody interactions. AI systems integrated with *in vitro* platforms can rapidly screen potential antibody candidates and rank their binding affinities or neutralization potential. For instance, patient-on-a-chip systems combined with AI algorithms have been used to profile drug responses and develop predictive models of immunotoxicity (Barrett et al., 2023; Chopra et al., 2023). In venom research, these tools may enable virtual screening of antibody libraries against toxin epitope databases derived from genomic and proteomic studies. As these computational pipelines mature, they promise a scalable, cost-efficient framework for advancing antibody-based alternatives to animal testing.

## 6 Future perspectives for antibody-based strategies to replace animal testing in toxin research and regulation

### 6.1 Antibodies for precision target toxins

Antibodies enable the precise detection and neutralization of a wide range of toxins due to their high specificity and selectivity. Diagnostic immunoassays, such as ELISA, are used to identify bacterial toxins (e.g*., C. botulinum, Clostridium difficile, S. aureus*, and *C. diphtheriae*), fungal toxins (e.g., aflatoxins), and animal-derived venoms (Reverté et al., 2014; Zhu et al., 2014; Pöhlmann and Elßner, 2020).

Therapeutic-neutralizing antibodies are being developed against toxins such as botulinum toxin, diphtheria toxin, and snake venoms, given their high lethality and bioterrorism risks (Cenciarelli et al., 2019; Rasetti-Escargueil and Popoff, 2019). Accurate detection and timely neutralization with these therapeutic antibodies are crucial for public health preparedness and treatment. Antibody-based methods now enable animal-free assays for potency and toxicity, including ELISA-based systems, skin sensitization tests, and SNAP-25 cleavage-based assays for botulinum toxin (Nepal and Jeong, 2020; Gądarowska et al., 2022). While immunoassays offer faster, simpler, and more cost-effective alternatives to mouse lethality assays, they often lack the ability to measure the biological activity or toxic effects of functional proteins (Koh et al., 2015; El-Sayed et al., 2022).

High-quality antibodies optimized for quantification and assessment of biological activity enable broader application of antibody-based alternatives. Key considerations include selecting antigen regions critical for biological function, designing recombinant or peptide antigens that maintain immunogenicity, and developing detection formats suitable for specific applications (Dormitzer et al., 2008; Caradonna and Schmidt, 2021; Guarra and Colombo, 2023). Increasing attention is focused on animal-free antibody discovery technologies due to ethical considerations. These alternatives to traditional animal immunization comprise phage, yeast, ribosome, and microbial display platforms (Jones et al., 2016; Zhang, 2023), direct B cell isolation from peripheral blood (Pedrioli and Oxenius, 2021), and epitope-directed selection methods (Chen et al., 2020; Zhu et al., 2023). Developments in AI have enabled *in silico* antibody discovery, design, and optimization through the integration of protein structure prediction and *de novo* sequence generation (Jeong et al., 2022; Hie et al., 2024).

High-throughput, label-free platforms, such as BLI, SPR, flow cytometry, lab-on-a-chip, and PPI assays, enable precise and reproducible antibody characterization, surpassing traditional animal-based methods (Picollet-D’hahan et al., 2021; Chun et al., 2024; Vacca et al., 2024). Standardizing these technologies may accelerate the transition to animal-free testing paradigms that meet scientifically robust and regulatory standards.

### 6.2 Matters to consider when standardizing protocols

To gain regulatory and industrial acceptance, antibody-based alternatives to animal testing require methodological standardization and rigorous validation. Although immunoassays with antibodies are widely used for assessing drug and vaccine quality, they must undergo rigorous comparative studies with traditional animal-based protocols (Nepal and Jeong, 2020; Bas et al., 2021). Despite their high specificity and sensitivity, immunoassays often fail to fully capture toxin functions or the *in vivo* neutralization capacity of antitoxins. Therefore, complementary cell-based or biochemical methods are needed to better reflect *in vivo* efficacy (Guideline, 2005; Koh et al., 2015). Ultimately, new approaches must demonstrate comparable or superior accuracy, reproducibility, and biological relevance to qualify as a true alternative to animal testing (Griesinger et al., 2016).

A central requirement during standardization is alignment with international guidelines, such as those established by the OECD, ICH, or WHO. These frameworks articulate best practices for validation and outline criteria for assay acceptance, including robustness, transferability, and inter-laboratory reproducibility (Kandárová and Letašiová, 2011; GIVIMP, 2018). Peer-reviewed validation and assessment by recognized accreditation bodies are critical in legitimizing novel methodologies intended for regulatory implementation (Bruner et al., 1996; Hartung, 2007). The key stages of standardization for antibody-based alternatives, along with corresponding guidelines and authorities, are summarized in Table 2. Furthermore, institutional and economic barriers require careful consideration. The pharmaceutical sector has traditionally depended on established animal testing paradigms due to their historical regulatory precedence, cost-efficiency, and familiarity. Consequently, even scientifically valid alternatives may face resistance unless they demonstrate scalability, cost-effectiveneess, and ease of use (Doke and Dhawale, 2015; Kanďárová and Pôbiš, 2024).

**TABLE 2:** Standardization stages for antibody-based alternatives.

Stage	Key Activities	Relevant Guidelines or Authorities
1. Method Development	- Design assay (e.g., ELISA, SPR) based on intended toxin/antitoxin target - Select appropriate antibody type and antigen format (e.g., peptide, recombinant protein)	OECD TG, WHO GIVIMP
2. Analytical Validation	- Assess specificity, sensitivity, linearity, reproducibility - Optimize assay conditions across labs	ICH Q2 (R1), OECD Guidance Documents
3. Biological Relevance Check	- Demonstrate functional equivalence or superiority to animal-based methods - Compare outcomes to established animal test results	OECD Performance Standards (e.g., TG 492, TG 442C)
4. Inter-lab Reproducibility	- Perform multi-site validation studies - Include blinded samples and proficiency testing	OECD GD 34, EURL ECVAM Recommendations
5. Regulatory Submission	- Prepare dossier for method recognition or inclusion in guidelines - Collaborate with regulatory agencies for review	OECD, FDA, EMA, MFDS
6. Implementation & Dissemination	- Publish in peer-reviewed journals - Promote via training, workshops, and inclusion in regulatory frameworks	ICCVAM, PARERE, Tox21

Antibody-based assay development should prioritize scientific rigor, practical implementation, and regulatory compatibility. By creating methods that satisfy market requirements and international validation standards, researchers can accelerate the adoption of animal replacement strategies in public health and commercial domains (Bas et al., 2021; Kahrass et al., 2024).

### 6.3 Importance of antibodies in toxin research

In toxin-related research, antibodies are commonly employed for diagnostic and therapeutic purposes. In diagnostics, immunoassays with specific antibodies are used to identify harmful agents, such as food allergens, bacterial exotoxins, and fungal toxins (Janik et al., 2019; Féraudet Tarisse et al., 2021; Avril et al., 2022). For example, ELISA kits containing targeted antibodies are frequently utilized to monitor aflatoxins, BoNTs, and SEs in food and clinical samples (Zhu et al., 2014; Janik et al., 2019).

Neutralizing antitoxins are included in the WHO Model List of Essential Medicines for treating potentially lethal intoxications, such as botulism, diphtheria, and tetanus. However, conventional antitoxin therapies, often derived from animal plasma, carry a significant risk of adverse effects, including serum sickness, hypersensitivity reactions, and nephritis (Mayers et al., 2003; Pirazzini and Rossetto, 2017). The development of monoclonal antibodies as therapeutics offers a more targeted and safer alternative. Examples include raxibacumab (approved for anthrax) and bezlotoxumab (approved for *Clostridioides difficile* infection), both approved by the US FDA and representing milestones in antibody-based antitoxin therapies (Tsai and Morris, 2015; Kufel et al., 2017). Beyond these examples, a broader range of antibody-based therapeutics for toxin neutralization at different development stages is summarized in Table 3.

**TABLE 3:** Antibody-based therapeutics for toxin neutralization.

Toxin Target	Antibody Name	Antigen Target	Application/Indication	Development Stage	References
*Bacillus anthracis* toxin	Raxibacumab	Protective Antigen (PA)	Inhalational anthrax treatment	FDA-approved (2012)	Kufel et al. (2017), Tsai and Morris (2015)
*Clostridioides difficile* toxin B	Bezlotoxumab	Toxin B	Prevention of recurrent *C. difficile* infection	FDA-approved (2016)	Tsai and Morris (2015), Wu et al. (2022b)
*Botulinum neurotoxin A*	XOMA 3AB, BI-CFPAB	BoNT/A light and heavy chains	Botulism treatment and biodefense	Preclinical/Investigational	Rasetti-Escargueil and Popoff (2019)
*Diphtheria toxin*	S315	Receptor-binding domain of diphtheria toxin	Diphtheria treatment	Preclinical	Alvarenga et al. (2014)
Snake venom (multiple species)	Human mAbs (various)	PLA2, metalloproteinases, neurotoxins	Antivenom replacement	Preclinical/Investigational	Pucca et al. (2019)
Pertussis toxin (*B. pertussis*)	BsAb-Ptx	Two non-overlapping epitopes on Ptx	Pertussis therapy, toxin neutralization	Preclinical	Wagner et al. (2016)
*Staphylococcus aureus* α-toxin	MEDI4893	α-hemolysin (Hla)	Prevention of *S. aureus* pneumonia	Clinical Trials (Ph2)	Tabor et al. (2016)

In the context of animal replacement, antibodies serve as tools for detecting toxins and are pivotal in developing non-animal-based potency assays for antitoxins. For example, neutralizing antibodies specific to BoNTs have been applied in ELISA-based assays to replace mouse lethality tests, demonstrating accuracy and reproducibility (Nepal and Jeong, 2020; Gdoura et al., 2022). Therapeutic antibodies targeting snake venoms are being investigated to substitute conventional equine-derived antivenoms, with promising efficacy and reduced immunogenicity (Pucca et al., 2019).

Although therapeutic antibody production has traditionally relied on animal immunization, ethical considerations have prompted the adoption of non-animal alternatives, such as phage display, yeast and ribosome display, and single B cell cloning from human peripheral blood mononuclear cells (Jones et al., 2016; Uchański et al., 2019; Pedrioli and Oxenius, 2021; Zhang, 2023; Porebski et al., 2024). The use of AI-assisted *in silico* antibody design and structural optimization is demonstrating potential to accelerate development while reducing reliance on animal models (Watson et al., 2023; Hie et al., 2024). Additionally, antibody-based analyses can be performed using high-throughput technologies such as SPR, BLI, flow cytometry, and lab-on-a-chip systems. These platforms facilitate rapid, quantitative, and reproducible data generation, contributing to the standardization of assays and minimizing operator-dependent variability (Picollet-D’hahan et al., 2021; Chun et al., 2024). As these tools become increasingly integrated with MPSs and machine learning algorithms, the capacity to functionally evaluate toxin activity and antitoxin efficacy without the need for animal testing will be substantially enhanced.

Antibodies are utilized as analytical and therapeutic tools in toxin research and are foundational in the development of animal-free testing strategies. Incorporating antibodies into high-precision immunoassays and computational workflows may influence approaches to toxin detection, characterization, and neutralization in preclinical research and public health preparedness.

## 7 Conclusion

Antibody-based technologies are becoming central to animal-free testing strategies within toxin research. Their use in diagnostic assays, neutralization studies, and therapeutic development highlights their scientific and translational value. Supported by advances in molecular engineering, MPSs, and AI-based modeling, these approaches have the potential to replace animal models with scalable and human-relevant platforms. Achieving this requires robust validation frameworks and consistent international standards. Integrating antibody-based tools with emerging *in vitro* models and computational methods can provide reproducible, ethical, and scientifically grounded alternatives for toxicology, aligning with the principles of the 3Rs.
